# Genomic research, publics and experts in Latin America: Nation, race and body

**DOI:** 10.1177/0306312715623108

**Published:** 2015-12

**Authors:** Peter Wade, Carlos López-Beltrán, Eduardo Restrepo, Ricardo Ventura Santos

**Affiliations:** Social Anthropology, School of Social Sciences, University of Manchester, Manchester, UK; Instituto de Investigaciones Filosóficas, Universidad Nacional Autónoma de México, Mexico City, Mexico; Departamento de Estudios Culturales, Pontificia Universidad Javeriana, Bogotá, Colombia; Fundacão Oswaldo Cruz – Escola Nacional de Saúde Pública, Rio de Janeiro, Brazil

**Keywords:** forensics, genomics, heredity, nation, public understanding of science, race

## Abstract

The articles in this issue highlight contributions that studies of Latin America can make to wider debates about the effects of genomic science on public ideas about race and nation. We argue that current ideas about the power of genomics to transfigure and transform existing ways of thinking about human diversity are often overstated. If a range of social contexts are examined, the effects are uneven. Our data show that genomic knowledge can unsettle and reinforce ideas of nation and race; it can be both banal and highly politicized. In this introduction, we outline concepts of genetic knowledge in society; theories of genetics, nation and race; approaches to public understandings of science; and the Latin American contexts of transnational ideas of nation and race.

This collection of articles presents findings from an interdisciplinary project that explored how knowledge generated by genetic research in Brazil, Colombia and Mexico circulated beyond the context of the research labs themselves, among diverse publics and research users. Our project was specifically interested in how research on genetic ancestry and genetic admixture – usually phrased in terms of European, African and Amerindian ancestries – might shape people’s ideas about the nation and its diversity, especially in relation to ‘race’ (including heredity and appearance), both at a collective and individual level. In some contexts, genetic scientists have linked their research findings explicitly to questions of the nation, national identity and health, racial–ethnic diversity and (anti-)racism, stimulating public debate on these issues and often influencing public policies.

Some approaches to the impact of genetic science on national and racial identities stress new forms of genetic citizenship and the transformative possibilities of ‘geneticization’. Other work argues that some genetic research re-inscribes, in a genetic idiom, existing racialized categories. Our results challenge simple one-directional processes of change, instead conveying the highly contested terrain on which this genetic knowledge works and the uneven impacts it has. Our work also conveys the importance of the nation as one of discursive frames within which these processes and categories take on meaning.

Latin America is a little-explored region for addressing these questions: the existing literature focuses mainly on the United States and Europe. In Latin America, with its national identities based on *mestizaje or mestiçagem* (racial and cultural mixture), race is often an absent presence – an everyday referent and concern and yet frequently disguised or seen as insignificant because of the dominance of *mestizos* (cf. M’charek et al., 2014a). National identities in the region are sometimes debated in terms of health – increasing rates of obesity and diabetes are sometimes described as national problems – which may be linked to the indigenous ancestry of mestizos. This makes the region interesting for exploring the social effects of genetic knowledge about nation and race. Latin American populations have long been subject to genetic analysis, yet the recent impacts of such analysis on ideas about the nation, its diversity and its past and future have barely been studied.

Our project involved three interdisciplinary and international teams carrying out in-depth research in each of Mexico, Colombia and Brazil over a total of 3 years.^1^ Researchers from cultural anthropology, sociology, biological anthropology and history of science participated in the project and give the articles in this Special Issue a broad disciplinary base.

## Race and genomics

New genomic knowledge may be reshaping social life through processes of ‘geneticization’, ‘biologization’ and the emergence of ‘biosociality’ and ‘biological citizenship’ (Franklin, 2003; Pálsson, 2007; Rabinow, 1992; Rose, 2007). The consequences of these processes are varied, not unidirectional and are still being explored.

There is no consensus on the question of whether new genomic knowledge re-inscribes race or not.^2^ On one hand, genomics can undermine the concept of race in very radical ways, by confirming the long-standing claim that biological race is a myth: because human biological variation cannot be divided up into meaningful categories that resemble what have, in previous science or in common sense, been taken as ‘races’.^3^ On the other hand, the sequencing of the human genome has ‘renewed and strengthened interest in biological differences between racial and ethnic populations, as genetic variants associated with disease susceptibility, environmental response, and drug metabolism are identified, and frequencies of these variants in different populations reported’ (Foster and Sharp, 2002: 845). Some geneticists argue that ‘racial and ethnic groups do differ from each other genetically’ due to geographically structured patterns of mating and reproduction and that these differences have ‘biologic implications’, including susceptibility to disease (Burchard et al., 2003: 1171).

Others do not talk in terms of racial groups, but refer instead to biogeographical or ancestral populations. Nevertheless, critics remark that these ‘populations’ seem to correspond to the ‘races’ that appear in old racial science, in some of the new genomics or in common-sense classifications: they divide people into familiar categories such as Africans, Europeans, Asians and Native Americans (Nash, 2015). Palmié (2012) states,

No matter then how much practitioners of genomics may protest their nonbelief in the biological existence of ‘races’ and tend to define their samples in terms of ‘biogeographical variation’ or ‘continental ancestries’, as soon as the findings thus produced are translated back into the language in which the question [e.g. about ancestry or origins] they are supposed to answer was originally formulated, we are back in the thoroughly racialized social worlds all of us – including molecular biologists and population geneticists – inhabit day in, day out. (p. 196)

The use of ‘population’ also can be problematic. It is common practice in genomic projects exploring human diversity to start with socially defined populations as sampling units (Gannett, 2001; Pálsson, 2007: Chapter 7; Reardon, 2005: 77–78): the practice makes cultural categories ‘appear to be genetic units; indeed it would *make* them genetic units’ (Marks, 2003: 203). If the cultural categories are already racialized ones, then race can be reproduced in the process, although what we might call second-order racialization (involving mestizaje) produces a complex form of reproduction.

Abu El-Haj argues that genomics is different from the old racial science because the biological markers it uses to differentiate geographically located genetic ancestries are often located in ‘junk’ DNA and do not shape phenotypical differences. Yet in another way, there is a ‘fundamental continuity between race science and anthropological genetics’, which rests on the assumption that ‘who we are collectively and individually is given by and legible in biological data which testifies to our origins, our pasts, to who we really are’ (Abu El-Haj, 2012: 23, 221).

A first phase of our project focused on genetic scientists’ production of knowledge (Wade et al., 2014b). We found that race had an ambiguous status in their research. Geneticists were all anti-racist, and they often (although not always) took an explicitly anti-racial stance, emphasizing that race was not a valid biological category. Nevertheless, geneticists reiterated foundational categories – African, European and Amerindian – when talking about the ancestries of Latin American populations. These were labels for ancestral populations or genetic ancestries, but the constant reiteration of them and the use of them to organize and display data suggested an overlap between social and genetic profiles, implying a genetic basis to familiar racialized categories and their mixtures. Genomic research projects also tended to reinforce existing social distinctions between populations within the nation perceived as ‘indigenous’, ‘Black’ and ‘mestizo’, by treating samples from these socially identified populations as separate.

Key to many arguments about how genomics re-inscribes race is the idea that geneticists’ findings have this unintended effect when they are translated into a more everyday language: ‘ancestry categories are often read as race and ethnicity’ by ‘other researchers, members of the media, and members of the general public’ (Fujimura and Rajagopalan, 2011: 23). For Palmié (2012), though, there is no simple division between expert geneticists and uninformed publics: the language into which genetic data are translated is the language in which the questions genetics seeks to answer were formulated to begin with – in this case questions of origins, relatedness, belonging, similarity and difference, which concern geneticists and publics alike. This indicates that while geneticists and lay people are separated by differences in technical expertise and knowledge, they also share some concepts (or language) and that the ‘same’ concepts circulate through different, but overlapping, domains of knowledge, where they acquire different meanings or serve different purposes. This suggests that while lack of technical knowledge may facilitate the reading of race into genetics, it is also the case that, as Marks (2003) says, geneticists’ practices may not only make social populations *appear* as genetic units but actually *make* them into genetic units. It is not just a question, then, of lay publics seeing race ‘by mistake’. We will explore this further below.

## Nations

In the first phase of our project, we found that genomic science tended to take for granted the nation as an obvious frame within which to conduct genomic projects that mapped genetic diversity, thus implicitly reproducing the nation as a (diverse) genetic community. The concept of the nation has not figured large in the literature on race and genomics, despite the well-known connections between ideologies of race and nation (Balibar, 1991; Taylor, 2011). While the nation appears in discussions about genomic patrimony and sovereignty (Benjamin, 2009; Rabinow, 1999), in studies of biobanks and national databases (Fortun, 2008; Hinterberger, 2012b; Pálsson, 2007) and in concepts of biological citizenship (Heath et al., 2004; Rose and Novas, 2005; Taussig, 2009), it has not been much explored as a racialized ‘imagined community’, the identity and diversity of which might be shaped, among different sectors of the public, by genetic knowledge produced about its constitutive populations (but see Hinterberger, 2012a; Kohli-Laven, 2012; M’charek et al., 2014b; Nash, 2013).^4^ In our research, we found that, given the close imbrication of race, mestizaje and nation in Latin America, the nation was a key frame for thinking about racial difference in genetic terms (Wade et al., 2014a).

In the newly independent Latin America of the 19th century, elites were preoccupied with the presence of indigenous, Black and mixed (mestizo) people. Given that the nations seen as modern and progressive in the world were perceived as either being mainly White or having highly segregated societies in which Whites were clearly differentiated from all non-Whites (including mestizos), Latin American elites were worried about the possibilities for progress in their countries. The racial science being developed in Europe and North America gave further grounds for pessimism, as it defined Black and indigenous populations as inferior and racial mixture as degenerative. However, the idea that mixture could create a more democratic society was soon mooted by some Latin American intellectuals. Others emphasized a form of eugenics that privileged the role of social hygiene, education and health in improving the population, playing down biological determinism.

There was variety in the way race and nation were interwoven in the region, depending in part on the demography and history of the country and influencing how blackness, indigeneity, whiteness and mixedness were configured in national ideologies. For example, many countries favoured European immigration to ‘improve the race’, but only some countries, such as Argentina and Brazil, attracted large numbers of migrants. This helped Argentinian elites to construct their country as a rather White exception to the Latin American rule. In Brazil and Mexico especially, and to a lesser extent in other countries, such as Colombia, during the early decades of the 20th century, elites developed a populist concept of the mestizo as a national icon and mestizaje or mestiçagem as the historical process that had created distinctive nations that could occupy a proud place in the world. In many nations, the African contribution to mestizaje was downplayed or ignored, but where it was acknowledged in some form – for example, in Brazil and to a lesser extent in Colombia – mestizaje allowed elites to claim that their nation was more racially tolerant and democratic than some reputedly more modern countries (Appelbaum et al., 2003; De la Cadena, 2007; Graham, 1990; Stepan, 1991; Wade, 2010).

In much of the region, the concept of race became seen – by national elites and by social scientists – as less important for understanding society than it was in, say, the United States. Ethnicity (understood as cultural difference) and culture itself seemed more relevant concepts in societies where the difference between an *indio* (indigenous person) and a mestizo was based mainly on language, clothing, place of residence and behaviour. Still, the term *raza* circulated – and circulates today – in everyday circles to refer to categories of people identified by various criteria, including colour (*la raza negra*) and national–ethnic origin (*la raza mexicana*) (Hartigan, 2013; Wade, 2010). In several countries, the ‘discovery’ of the Americas is commemorated by the so-called *Día de la Raza* (Rodríguez, 2004). In that sense, race is an absent presence in the region: it may be denied or downplayed, but it is also still there.

Since about 1990, many Latin American countries have pursued multiculturalist reforms, recognizing ethnic diversity in fields such as constitutional reforms, land rights, political representation, education and health policy. These reforms have highlighted indigenous and Afro-descendant groups as deserving special attention, breaking the previous trend to see them as inputs into a process of nation-building based on mestizaje and modernization. There has also been an increased but regionally uneven attention to racism and racial inequality as social problems.

Still, the idea that mestizaje is an underlying national reality has not been submerged by multiculturalism. On the contrary, the centrality of ideas of mixture to national identity is fundamental to understanding genomic projects in Latin America. Mestizo populations are genomic objects that circulate in an international science world: ‘admixed populations’ are a useful resource for certain kinds of enquiry, such as ‘admixture mapping’, a tool for localizing disease-causing genetic variants (Darvasi and Shifman, 2005; Wang et al., 2008). Meanwhile, geneticists – and their publics – are also very interested in knowledge about the nation and its origins, mixedness and current diversity.

## Publics

In this Special Issue, we focus on how knowledge produced by genomic projects circulates beyond research laboratories. Work on the circulation of genetic knowledge beyond labs often focuses on patient groups or categories of people with very direct, often health-related, interests in ‘biosociality’ (Heath et al., 2004; Rose, 2007; Rose and Novas, 2005; Taussig, 2009); less research has looked at how more ‘general publics’ interpret genetic knowledge (Condit, 1999; Condit et al., 2002, 2004; Jacob, 2012). Analyses may not actually deal with members of ‘the public’ even when they are arguing that genetics re-creates race in public domains such as health care (Duster, 2006; Kahn, 2013; Roberts, 2011). These studies generally highlight the ‘molecular reinscription of race’, to use Duster’s phrase, although some argue that the effect of genetic knowledge on people – usually patients or prospective patients – is to recast race as a risk factor, which they should take into account in projects of self-care and prudent self-actualization, rather than as a simple determinant (Abu El-Haj, 2007; Rose, 2007: 155–186).

Other research has focused on media translations (often by science journalists) of technical findings into a more accessible form, perhaps in a way that seems conducive to reifications of race and ethnicity by a notional ‘public’ (Campbell, 2007; Fujimura and Rajagopalan, 2011; Lehrman, 2008; Nash, 2012, 2013; Nelkin and Lindee, 1995b). These studies overlap with other research that has concentrated on commercial genetic technologies, focusing on the companies that provide these services – for example, how they present genetic data on their websites and publications – and/or the people who have chosen to explore their genealogies using such companies; the blogs attached to ancestry testing websites are often used as a source of data about people’s responses (Abu El-Haj, 2012: Chapter 4; Nelson, 2008a, 2008b; Schramm, 2012; Sommer, 2011, 2012; TallBear, 2008).

Where these studies deal with the reactions of ‘ordinary people’, the picture they paint is of ambivalence and the strategic use of genetic information in reckoning about race and ethnicity. People both attribute to genetics a privileged access to the truth – DNA uncovers ‘reality’ – and yet may also be sceptical about scientific claims. People will strategically combine the ‘genetics facts’ with their own ideas about who they are, their origins and their belonging to create narratives that make sense to them and express their own life projects. African American and Black British and UK consumers of genetic ancestry tests, for example, combine genetic data and other kinds of genealogical information to construct ‘meaningful biographical narratives’; DNA evidence is not always accepted as definitive, and the geneticization of race and ethnicity that is the ‘basic logic’ of genetic ancestry testing ‘is not necessarily its inexorable outcome’ (Nelson, 2008a: 761). Native American high school students, when confronted with DNA evidence suggesting an ancient male skeleton – Kennewick Man – found in US soil was not conclusively ‘Native American’ in terms of his DNA, were at first inclined to cede authority to the scientists: ‘if they say so …’. After some guidance from older mentors, who presented post-colonial perspectives that valued indigenous knowledge as highly as scientific accounts, the students were more inclined to challenge the DNA evidence, without throwing it out of court (Jacob, 2012). Focus group research in the United States indicates that the circulation of information about genetics does not simply lead people to explain in terms of genetics the traits that they perceive as linked to race: ‘personal will, culture, and faith in God’ continue to play important roles in people’s ideas about race, health and behaviour (Condit et al., 2004: 269).

When genetics enters the legal process, as providing evidence to settle claims about rights and identifications, the results are also ambivalent. In the domain of forensics – and especially in the way forensic analysis is presented by the media – there seem to be strong tendencies towards the biological reification of race. Forensic specialists and the police are keen on the possibility that useful descriptions of an individual’s racialized appearance can be reliably deduced from his or her genetic ancestry – and the press often take them at their word (M’charek, 2008; Sankar, 2012). In the United States, race continues to be used as a parameter in DNA matching, despite the fact that its previous relevance for improving the accuracy of probability ratios for matches has been superseded by technical advances (Kahn, 2012). On the other hand, when ancestry tests are used by plaintiffs seeking to claim rights, courts may not accept their relevance. For example, in a 2004 case, ‘Black Indians’ claiming membership of the Cherokee Nation used DNA evidence showing they had Native American ancestry (despite looking ‘Black’). This was rejected by the Cherokee leaders who stuck to the government criteria that admitted as members only direct descendants of individuals originally listed as ‘Indians’ by late 19th-century government bureaucrats (Hamilton, 2012).

All this suggests that people’s ideas about race and ethnicity are not undergoing a one-way process of ‘geneticization’ (Wade, 2007). Instead, a more uneven and complex process is at work, in which people strategically adapt aspects of genetic knowledge in ways that may be more or less deterministic. The transformative power of genetic knowledge is, in the end, rather uncertain: to what extent does it recast existing ideas, albeit in complex ways and not necessarily towards fixity and determinism, and to what extent do existing ideas simply scavenge new genetic data and deploy them within familiar frameworks, which may already contain strong possibilities for determinism? To what extent is it true that identity and race, when ‘rewritten at the genomic level, visualized through a molecular optic’ are ‘both transformed’ (Rose, 2007: 179)? Going to a more fundamental level, should we be asking ‘what kind of human is brought into being via genomic analysis’ in order to grasp how human diversity is being construed, as genomics ‘reconfigures both nature and society’ (Reardon, 2005: 16)?

We argue that the power of genomics to transform and reconfigure race, identity and humanity is very uneven, particularly when genomic knowledge circulates in lay circles; in some contexts, the language of transformation and reconfiguration may overstate the case. A good example of the complexities involved is Kohli-Laven’s (2012) exploration of what happened to French-Canadian identity and ideas about health when genetic epidemiology researchers took over a digitized database of Church registers going back 400 years. When the registers were first digitized, university demographers used surnames, as recorded by priests, to indicate racial or ancestral origin. This created, or reproduced, a clear cultural division between Whites and Native Americans because Church policy was to assign French names to natives seen as acculturated. When the genetics unit used the database to research epidemiological questions, names became indicators of ‘not just French social, moral and religious status, but of “French biology”’ (Kohli-Laven, 2012: 192). French-Canadians appeared as a pure lineage.

For local people thinking about the incidence of certain diseases in their families, the idea of in-marrying and in-breeding as a possible explanation already existed, but it was located mainly at the level of the family. Geneticists’ and doctors’ explanations of population-level endogamy, which created founder effects that increased the risk of particular diseases, relocated (some) people’s narratives to the level of ‘French-Canadians’ as a collective. As one woman, founder of a patient association, put it, ‘All of a sudden we knew it was not us [our family and its genealogy] but our population as a whole. We never took an interest in genealogy after that’ (Kohli-Laven, 2012: 196). Racialized ideas about the nation already existed, but they were re-crafted in the light of new genetic information. Meanwhile, the genetic information – although generated by scientific protocols and not a simple effect of pre-formed ideologies – had itself been shaped by these ideas, insofar as Church records were taken as unproblematic evidence of ancestral origin.

It seems an overstatement here to talk of genomics as ‘transforming’ identity and race, and while ‘reconfiguration’ is closer to the mark, it is a moot point whether the ‘kind of human’ that is brought into being in this case is noticeably different. Rather, new variations on familiar themes are potentiated. People had already considered themselves as human members of a collective, who shared both history and genealogy; with genomics, that concept of the human remains fundamentally the same but is relocated to permit specific problems that hitherto had been thought of in terms of family genealogy to be explained in terms of the larger collective. This thread of continuity is also spun by the circular, rather than one-way, traffic occurring between genomics and identity/race: genomics often takes its categorizations from the social realm in the first place – as the demographers did in reading the Church records – and thus tends to re-inscribe them, albeit now with slightly different affordances.

Here, we take a co-productionist perspective, in which neither ‘science’ nor ‘society’ is given a privileged role and instead natural and social orderings are seen as linked in complex mutual accommodations. New scientific objects and assemblages (e.g. the ‘map of the genome of Mexican populations’) shape and are shaped by social orderings (Jasanoff, 2004; Reardon, 2005). The approach is especially relevant to categories such as ‘nation’ and ‘race’, which have long acted as boundary objects in scientific and social classifications (Bowker and Star, 1999). Wynne’s idea that the ‘imagined publics’ that scientists see themselves as addressing, albeit indirectly, may shape their scientific practices (Wynne, 2005) is particularly apposite when geneticists deal with ‘mestizos’ or ‘Afro-descendants’ and ‘nations’, which are already public concepts. The point is that genomic knowledge does not just emerge from research labs and affect the wider society: key categories – a specific population label or a concept such as ‘nation’ – are basic to the construction of the genomic knowledge in the first place. And such categories are themselves often products of long-term interactions between science and society, as historians, physical anthropologists, geneticists, demographers, bureaucrats, politicians and ‘ordinary people’ have participated, perhaps in conflicting ways, in establishing and defining the identities of populations, whether local or national.

This approach to the ‘public understanding of science’ avoids the old ‘deficit model’, which posited, on one side, an ignorant public and, on the other, a body of informed scientists, who had an ethical and citizenly obligation to be ‘transparent’ and educate the public. Not only can ‘the public’ not be viewed as a passive recipient, it must also be seen as actively involved in the construction of scientific knowledge as it circulates in society (Edwards, 2002; Irwin, 2001; Jenkins, 1999; Wynne, 1992). Our research reinforces the idea that the engagement by various publics with genomics involves the strategic mobilization of a variety of ideas and experiences to manage scientific knowledge about nation, race and genetics.

## Latin America

Little research has addressed these issues of the circulation of genetic information in public domains in Latin America (but see Díaz del Castillo Hernández et al., 2012; Gaspar Neto and Santos, 2011; Kent, 2013; Santos et al., 2009). The Latin American context is interesting because, as noted above, multiculturalist reforms enacted since 1990 have generated public debate about national identity and diversity, prefigured by earlier critiques of mestizaje as a homogenizing and authoritarian ideology. The concept of race – multifaceted and often semi-visible in the region – has gained some traction, principally through concerns about racism, but also as a (contested) way of talking about diversity within the nation (Arias and Restrepo, 2010). Genomic research has entered these debates through the media, including through the participation of geneticists in public dissemination.

It is useful to give an overview of the genomic research that has circulated most widely in the public domains we studied. The Mexican National Institute of Genomic Medicine (INMEGEN) carried out research to create a ‘genomic map of the Mexicans’, published in 2009. INMEGEN built upon a long tradition of Mexican human medical population genetics, which made the mestizo a central object of research (López-Beltrán and García Deister, 2013). The genomic map project was part of a national medical genomics agenda based on the idea that Mexicans need medical treatments tailored to their genetic characteristics as mestizos, rather than ones supplied by foreign multinationals (García-Deister and López-Beltrán, 2015; Kent et al., 2015). INMEGEN actively publicized its national genetic database project, along with its account of diversity in the genetic ancestry of Mexicans.

Scientists at Brazil’s Federal University of Minas Gerais have done research on the ancestry of Brazilians, highlighting that many ‘Whites’ have African and Amerindian ancestry, while many ‘Blacks’ have high levels of European ancestry. Genetic data have been used to confirm the existing idea that the Brazilian population was formed on the basis of the mixture of European men with indigenous and African women. The geneticist Sérgio Pena, in particular, has vigorously challenged the validity of ‘race’ as a concept and has engaged in political debates about the legitimacy and practicality of race-based affirmative action programmes for Brazil as a mixed nation (Kent et al., 2015; Kent and Wade, 2015).

In Colombia, genetic research tends to highlight regional differences within the nation, linking them to racial diversity in a way that meshes with common narratives about Colombia as a country of distinctive regions and which is subject to (often violent) fragmentation (Olarte Sierra and Díaz del Castillo Hernández, 2013). One geneticist has published popular science books, relating genetic data to issues of nation, race and mestizaje (Yunis Turbay, 2009). In other contexts, genetic research has been used to establish standardized technologies for government forensic technicians, identifying disappeared relatives in the context of violence, understood as a national malaise (Díaz del Castillo Hernández et al., 2012).

These national scenarios present fascinating opportunities for exploring how genetic knowledge circulates beyond laboratories in the wider society (and back into the labs). This is a region where concepts of race are flexible and ‘culturalized’ and are both present and absent; a region where multiculturalist reforms are transforming debates about the nation and its diversity, raising the profile of differences construed as ethnic and, at times, racial; a region where there are debates about the health of the nation, in the context of rising rates of obesity and diabetes, and high levels of violence. Recently, genetic data about gendered processes of mestizaje, diversity within the nation (usually phrased in terms of ancestries), health and pathology, and the question of biological races have, to varying extents in the three countries, circulated in the public domain, where issues of diversity and nation are common currency. Has the emergence of a genetic discourse about these issues geneticized and/or otherwise ‘transformed’ people’s ideas about race and nation and, more broadly, human diversity, especially in a context in which ideas about race were anyway so ambiguous and absently present?

## Mexico, Brazil, Colombia: The power of genomic knowledge?

Our project explored the circulation of genomic knowledge about ancestry and human diversity in a number of contexts – not only among lay publics (mainly focus groups and interviews with university students, plus some Black social movement activists and volunteers for a genomic research project) but also among other kinds of specialized experts, such as forensic technicians, public health policy-makers and medics, who constitute specific kinds of ‘publics’ that use the specialized genetic knowledge produced by the genetic scientists.

For Mexico, our project explored how scientific genetic knowledge produced about mestizos (and indigenous people) has entered into the wider public sphere and whether it has shaped people’s ideas about the nation, ‘race’, ancestries and bodies, and health. There is little doubt that the state-backed project of mapping ‘Mexican genomes’ provided a language for defining the nation in biological terms. The common-sense equation of *la raza mexicana* with ‘mestizos’ is a complex one. It is a long-standing official representation, with connotations ranging from tough masculinity to the utopian idea of ‘the cosmic race’ (Irwin, 2003; López-Beltrán et al., 2014a; Vasconcelos, 1997 [1925]). Yet, it is filled with ambivalence, shot through with a post-colonial anxiety about inferiority and about being the products of conquest and rape (Paz, 1950). Calling oneself mestizo is not the first response of most Mexicans, when asked to identify in generic terms – they are likely to say ‘I am Mexican’, using ‘mestizo’ as a descriptor only in particular contexts. Yet the idea that the nation was formed from the diverse mixture of (mainly) Europeans and indigenous peoples is taken for granted and the Mexican genome project powerfully endorsed that assumption, providing a language of genetic ancestry for articulating it.

In addition, genomic science is being promoted as an important solution for the nation’s perceived pathologies of health (levels of obesity and diabetes) and escalating violence. In Mexico as elsewhere, medical genomic research aims to provide ways to help tackle ‘complex disorders’ such as diabetes, while forensic genetic technologies identify individuals as a means to bring perpetrators to justice and to identify bodies for the relatives of the disappeared. As García-Deister and López-Beltrán (2015) show in their article in this issue, the trajectories of medical and forensic genetics have been rather different, reflecting different ways stakeholders have formed these assemblages. Medical genomics has followed a top-down trajectory, driven by the state and the medical establishment, with diverse interests converging on the foundation of a national institute of genomic medicine as a way to promote interests in public health, national sovereignty, biocapital, pharmaceutical industries and so on. Forensic genomics is emerging in a less top-down way, being driven in part by the demands of citizen groups that press for reliable and transparent protocols for the identification of bodies killed in a context in which the perpetrators of violence may be perceived as linked to state security forces.

Whatever the differences between medical and forensic genomics in Mexico, they both clearly use a language of genetics to articulate notions of the nation, belonging and citizenship. Medical genomics assumes a common citizenship rooted in shared genetic ancestry – and thus a sharing, in some degree, of disease risk at the collective level (as in the French-Canadian case). In forensics, it is about letting the ‘truth’ flow unimpeded through the proper channels: the ‘reality’ that is understood to be the inherent potential of genetic identification is subject to distortion and corruption by faulty protocols and blockages caused, intentionally or otherwise, by institutional means. This is not a question of collective belonging to a nation or ‘race’, but of collective rights to information and, ultimately, justice and redress. DNA can release truth in a highly potent way (i.e. full of potential) because the results it generates are seen as (almost) incontrovertible and its presence is so ubiquitous (in all tissue, not just teeth or bones). But the truth released needs the proper institutional context to flow in channels that lead to justice. This is a form of genetic citizenship in the sense that rights are perceived by certain people – those seeking missing relatives – to be potentiated through DNA.

However, focus groups held in Mexico with university students and others who had little direct interest in genetics, DNA matching or ancestry tests indicated a rather different scenario, which heavily qualifies any notion of a generalized process of geneticization or the expansion of genetic citizenship (López-Beltrán et al., 2014b). These groups gave little evidence that their notions of race, ancestry, heredity and body are being transformed by the circulation of genomic data. This was even true of individuals who had participated in a scientific research project in which they were given explanations about genetic ancestry and were required to estimate their own ancestry in racialized terms. A genetic language for thinking about heredity, identity and belonging did not gain much traction in people’s minds, despite people’s acquaintance with the gene as ‘icon’ (Nelkin and Lindee, 1995a). There was a greater resonance when considering personal health issues, because a predisposition to certain disorders could increasingly be seen as linked to one’s biological condition as a mestizo, a condition publicized by public health campaigns and the Mexican genomic diversity project. But even then focus group participants avoided a deterministic approach and emphasized lifestyle and diet. Overall, how genomics ‘transformed’ or ‘reconfigured’ people’s ideas about nation, race, belonging and health depended strongly on context: for some people – usually those with a vested interest – genomics could have a powerful effect; for others, genomic knowledge was one more element in a broad toolkit of established ways of thinking about those things, an element that did not significantly reconfigure the toolkit.

In Brazil, race is, on one hand, very publicly present: censuses have (with some periodic breaks) asked people to identify their ‘colour’ since the late 19th century; in 1991, the question changed to one about ‘colour or race’. Since the mid-1990s, there has been official recognition that racism is a problem, leading to race-based affirmative action programmes in higher education and health policy and, in 2010, to a Statute on Racial Equality. On the other hand, race has been subject to explicit denial: at the same time that a colour question re-appeared in the 1940 census, the state had already started to represent Brazil as a ‘racial democracy’, an enduring image that, while it recognized racial inequality, subsumed this in class inequality and affirmed the relative insignificance of race in a society where many people are mixed. The introduction of race-based policies is often seen by critics as the imposition of foreign (usually US) values onto a Brazilian context unsuited to them. Critics argue that social policy should combat inequalities through race-blind measures, rather than highlighting racial categories (Fry et al., 2007; Guimarães, 2007; Htun, 2004; Telles, 2004). These debates about importance of race and racism keep the idea of race alive in the public arena. Meanwhile, the geneticist Sérgio Pena has publicly campaigned against race as a biological concept and as a concept appropriate to the Brazilian context. The issue is, then, how does the public circulation of genetic descriptions of Brazilian diversity and mixed ancestry shape these debates?

Our project explored the roles that genetic knowledge has played in discussions about race-based affirmative action and how Black social activists, in particular, deal with interventions by scientists that attempt to undermine affirmative action in higher education by adducing genetic data. We looked at Pena as a key figure in the way genetic knowledge is being actively used to reaffirm a representation of the nation as pervasively mixed, a place where globalized definitions of racial identity, perceived as rooted in the US experience, are seen as inappropriate and not in tune with what he has dubbed ‘*Homo brasilis*’.

Kent and Wade’s (2015) article in this issue illustrates a paradox in relation to ideas about the geneticization of society, race and nation. Pena has long argued that genetics conclusively shows that race does not exist as a valid biological concept; in this, he is at one with many other geneticists worldwide. He insists that Brazil, in particular, is a very mixed society in terms of genetic ancestry – something he and others have demonstrated with genetic data showing that self-identified ‘White’ Brazilians have indigenous and/or African genetic ancestry; that ‘Blacks’ and ‘Browns’ have plenty of European ancestry; and that most Brazilians bear the genetic imprint of early colonial mixing between European men and indigenous and African women. These data have been deployed to argue that race-based affirmative action health policies directed at ‘Black/Brown’ people might be misdirected, if they assume a health-relevant biological profile shared by ‘Blacks/Browns’. As Kent and Wade show, such data have been deployed by critics of racial quotas in higher education, who argue that these policies are inappropriate in a context in which no one is ‘really’ Black and everyone is ‘really’ mixed.

This attempted de-racialization of society can paradoxically imply a geneticization of society. One version of the argument is that races do not exist genetically, and least of all in Brazil, and that *therefore* they should not exist socially. Pena himself is cautious about making this leap from the objective to the normative. He says that science cannot define social policy (about affirmative action), but that policy-makers should be aware of the evidence about genetics and race, especially with regard to health policy, but also more generally (Pena and Bortolini, 2004). This was evidence that Pena gave to the 2010 Supreme Court hearings on the constitutionality of racial quotas. In effect, Pena advocated a Latourian modernity in which science and society were separate; at the same time – also like a true Latourian modern – he wanted to hybridize the two spheres by suggesting that policy-makers base their decisions on the order of nature, as revealed by science (Latour, 1993). The message is inescapable: genetic facts should be taken into consideration in the ordering of society. The message gained some traction in the public sphere: Kent and Wade recount that widely publicized DNA tests showing that the iconic Black musician, Neguinho da Beija Flor, had 67 percent European ancestry were used by some to criticize race-based affirmative action in higher education and the very idea of a ‘Black’ identity in Brazil.

In contrast, Black movement activists preferred a de-geneticization of society: if the ‘modern constitution’, in Latour’s (1993) terms, decreed that the social order should be separate from the natural one, then so be it. Genetics had no place in social policy, at least in relation to higher education; policemen did not ask for a DNA test before deciding to beat a ‘Black’ man; decisions about whether to re-racialize society by introducing racial quotas should be motivated by the facts of the social order (e.g. racial discrimination has been proven to exist), not facts about the natural order. Debates about racial quotas in higher education should take place without reference to genetics.

In the end, the Supreme Court, the government and the state decided that Brazilian society did need racial quotas; the genetic evidence was deemed irrelevant. The social order was de-geneticized in that genetics was not admitted as a force that should guide higher education policy, even if the language of genetics became widely available as a resource people could use to comment on issues of race and nation.

Yet, the outcome in Brazil is more complex and uneven than this. The article in this Special Issue comparing the Mexican and the Brazilian scenarios – symbolized by the images of the *genoma mexicano* and *Homo brasilis* (Kent et al., 2015) – indicates that, in both cases, genomic research projects produced powerful images of the nation as a genetic community – diverse, to be sure, and far from homogeneous, but a nation conceivable nonetheless in terms of its historic mixture of three distinct genetic ancestries (European, Amerindian and African). Such images were already powerfully entrenched in both countries – although the African element in Mexico was decidedly marginalized, despite recent multiculturalist attention to the so-called ‘third root’ – so this was not a case of genomics research ‘transforming’ race and identity in Brazil or Mexico. But neither is it a case of the de-geneticization that the Black activists were seeking to accomplish. It was, again, a case of genomics providing another element in the toolkit of ways to think about race and nation. State multiculturalism had weighed more heavily than genomic data in deciding on the affirmative action policies that went against the grain of the image of Brazil as a mestizo nation; yet that image, so powerfully engrained in common-sense, had not by any means been erased and now genomic data re-affirmed it, with the genetic idiom resonating with already existing ideas about genealogy and heredity.

Colombia is an interesting case because there has been less public debate involving genetic data, although genetic scientists have been active in producing knowledge about the country’s diversity and ancestry and some have also published popular books and/or been the subject of press coverage about their research (Barragán Duarte, 2007; Fog, 2006; Yunis Turbay, 2009). The article in this issue by Schwartz-Marín, Wade, Cruz-Santiago and Cárdenas shows how a specific genetic description of regional populations in the country became a standardized reference tool for establishing likelihood ratios in DNA matching, which was used by the state’s family welfare institute for paternity testing and by the state’s forensic science institute for identifying dead bodies and suspects (Schwartz-Marín et al., 2015). The forensic technicians who use this tool in everyday practice represent a specialized kind of ‘public’, and as with studies of the impact of genomics that focus on patient groups or specific legal cases, this needs to be borne in mind when assessing overall processes of geneticization, transformation and reconfiguration. But it is still an important area in which to assess how genomic research shapes images of race and nation.

The forensic tool in question was a research paper presenting four tables of allelic frequencies, based on samples taken from all over the country and clustered, by statistical means, into four regional categories, described without explicit reference to ‘race’ but in terms of the presence of ‘African descendants’, ‘mestizos’ and ‘an Amerindian component’ (Paredes et al., 2003). The research represented the nation as divided into racialized regions, following – and geneticizing – widespread existing ideas about the nation and its internal diversity. Colombia has long been seen as a country of racialized regions and the authors of this article derived their four basic regions from existing historical sources before finding that their genetic data fit the regional pattern. The tables became part of standard forensic practice, but the odd thing about them was that (a) regional differences in allelic frequencies were very small and did not indicate significant population differentiation, and (b) when technicians actually used the tables to generate probability ratios for a match, it made virtually no difference which table they used. The kinds of genetic markers used for forensic analysis are not good for differentiating in terms of biogeographical ancestry.

The insistence by Paredes and colleagues on regional difference is similar to what Kahn (2012) calls the ‘inertial power of race’ in the US legal system: he shows that although the statistical differences between the racial categories used in DNA matching have become legally irrelevant for purposes of establishing proof, they are still used, thus genetically reifying race. It seems likely that the idea of using region to organize DNA data in Colombia was following perceived international good practice, in this case the US practice of using racial categories to achieve ‘more accurate’ results. (If the Colombian researchers had had the relevant racial identity data, perhaps they would have used these instead, but they had to make do with regions.)

In Colombia, region and, indirectly, race were also being reified in genetic terms and then reproduced in everyday practice by forensic technicians. Even if they recognized that the difference between the four tables was irrelevant for determining a result, the regional–racial schema inscribed in them was already taken for granted, which gave it ‘inertial power’ and allowed the tables to form part of local habit. The process of geneticization involved here, however, was hardly transformative, precisely because the technicians could see that, with these genetic markers, there was no ‘real’ difference between the regions. Again, then, a genetic language became available for thinking about race, region and nation, but its traction for producing real change was limited by the circularity of the relation between the familiar and the new. Traction was also reduced by the fact that when a forensic DNA identification entered the courtroom, although it was explicitly associated with one regional table in the expert report, the regional–racial link disappeared because the technology was able to produce extremely high levels of probability of matching, whichever table was used.

As in Mexico, our Colombia team carried out focus groups and interviews with a range of people – mostly but not only university students – to assess whether geneticization of thinking about race and nation was taking place (Schwartz-Marín and Wade, 2015). Especially among students in the life sciences – and also among policemen and forensic specialists – there was a clear tendency to see Colombia as a country marked by the ‘regionalization of race’ and the ‘regionalization of genes’, to borrow terms used by geneticist Emilio Yunis Turbay (2009: 19); there was little problem in seeing region in biologizing terms. As with the forensic tables, however, it was hard to see recent genomic research on Colombian population diversity as having caused this: such views were already part of common-sense perceptions of the country. However, the fact that humanities students were much less likely to use this kind of biologizing and racializing language indicates the unevenness of views, apparently as a result of people’s intellectual backgrounds and training. Ideas about the ‘geneticization’ of society have to take this unevenness into account.

If some students were happy to dabble in biologizing and geneticizing idioms when talking of regions, when it came to more personal ponderings on identity, ancestry and heredity, people in general adopted a more flexible approach, emphasizing – as did Mexican respondents – that many different factors could play a role. They explained their own estimates of their racialized ancestry in terms of how they looked, what their surnames were and where they and their parents came from. They might see predilections and tastes as hereditary, as well as somatic features, and they might use a language of latency and manifestation or expression to account for discrepancies between the known ancestry and actual phenotype or behavioural tendencies of a person. But none of this is hard evidence of geneticization, as all these discursive elements have been part of widespread ways of understanding human (and animal) heredity for a long time (López-Beltrán, 2004; Tyler, 2007; Wade, 2002), added to which a basic language of genetics has been around for over 100 years and has entered into the toolbox of concepts people use to talk about heredity. As argued above, when talking of the ‘public understanding of genetics’, we are talking about the long-term circulation of ideas through different domains of expertise, rather than a one-way impact of genetic experts on lay people.

The Colombian focus group data included an interesting exercise in which people were asked to imagine their reactions to genetic ancestry tests that gave them very unlikely-looking results – for example, that a very light-skinned person had 90 percent African ancestry. Very striking was the fact that people mainly ceded authority to the test – ‘if the scientists say so ….’ Yet they would also then attempt to make that result intelligible in terms of what they already knew about themselves and their families. Like the Mexican respondents, these people were saying that the scientists might technically be right, but what mattered was how it made sense to them personally. The authority of genetics does seem to make a big impact on people – it is seen as giving access to the truth – but as Nelson (2008a: 762) says, the truth of *bios* (biological life) is always mediated by truth of bios (biographies).

## Conclusion

Our data show the very uneven and even contradictory impact of genetic knowledge on different publics. Widely seen as irrefutable and necessary – but only if operating in a transparent institutional environment, beyond the influences of political corruption and state malfeasance – genetic knowledge nonetheless lacks the power to transform or rewrite definitions of diversity, humanity, race and nation across a range of social contexts. Instead it tends to reinforce existing definitions, sometimes lending them particular force – with scientific veracity or the revelation of the invisible – but not transfiguring them. Genetic discourse itself does not simply reproduce concepts of race – for example, it undoes any notion of racial ‘type’, it multiplies definitions of ancestry (into uni-parental and bi-parental lineages) and it recasts ancestry into an abstract and precise-looking language of percentages instead of an idiom of genealogical proportions (full, half, quarter). But the way this discourse enters into broader realms of experience, outside the lab and the research paper, means transformative potentials are not fully realized and more familiar concepts of race and nation are often re-inscribed.

Yet, genomic knowledge can also provide the tools for defining both nation and race in altered ways – without transfiguring the underlying concepts. Thus, Mexico can be defined as a pathological nation, in a way that references the national population’s mestizo ancestry, as well as its eating habits. Or in Brazil, when DNA tests revealed the 67 percent European ancestry of Neguinho de Beija Flor (little Black man …) some commentators ironically re-named him Branquinho de Beija Flor (little White man …) – a barb that gained its force in the context of the heated debates about whether ‘Black’ recipients of race-based quota places at universities were ‘really’ Black. In short, genomic knowledge could unsettle and reinforce ideas of nation and race; it could be both banal and highly politicized. The end result is a great deal more complex than current ideas about the geneticization of society or the transformation and re-writing of identity and race.

## Notes

1. See http://www.socialsciences.manchester.ac.uk/subjects/social-anthropology/our-research/projects/race-genomics-and-mestizaje/. See also Wade et al. (2014).

2. Some of the relevant literature on this is cited in the articles in this Special Issue. See also Abu El-Haj (2007), Bliss (2012), Duster (2015), Fullwiley (2007, 2008), Koenig et al. (2008), Krimsky and Sloan (2011) and Whitmarsh and Jones (2010).

3. See, for example, Brown and Armelagos (2001), Dupré (2008), Lewontin (1972) and Reynolds and Lieberman (1996).

4. Abu El-Haj’s (2012) work on the search for Jewish origins is relevant here. See also Wade (2007).
